# A chromosome-level genome assembly of the orange wheat blossom midge, *Sitodiplosis mosellana* Géhin (Diptera: Cecidomyiidae) provides insights into the evolution of a detoxification system

**DOI:** 10.1093/g3journal/jkac161

**Published:** 2022-06-25

**Authors:** Zhongjun Gong, Tong Li, Jin Miao, Yun Duan, Yueli Jiang, Huiling Li, Pei Guo, Xueqin Wang, Jing Zhang, Yuqing Wu

**Affiliations:** Institute of Plant Protection, Henan Academy of Agricultural Sciences, Key Laboratory of Crop Pest Control of Henan Province, Key Laboratory of Crop Integrated Pest Management of the Southern of North China, Ministry of Agriculture of the People’s Republic of China, Zhengzhou 450002, P. R. China; Institute of Plant Protection, Henan Academy of Agricultural Sciences, Key Laboratory of Crop Pest Control of Henan Province, Key Laboratory of Crop Integrated Pest Management of the Southern of North China, Ministry of Agriculture of the People’s Republic of China, Zhengzhou 450002, P. R. China; Institute of Plant Protection, Henan Academy of Agricultural Sciences, Key Laboratory of Crop Pest Control of Henan Province, Key Laboratory of Crop Integrated Pest Management of the Southern of North China, Ministry of Agriculture of the People’s Republic of China, Zhengzhou 450002, P. R. China; Institute of Plant Protection, Henan Academy of Agricultural Sciences, Key Laboratory of Crop Pest Control of Henan Province, Key Laboratory of Crop Integrated Pest Management of the Southern of North China, Ministry of Agriculture of the People’s Republic of China, Zhengzhou 450002, P. R. China; Institute of Plant Protection, Henan Academy of Agricultural Sciences, Key Laboratory of Crop Pest Control of Henan Province, Key Laboratory of Crop Integrated Pest Management of the Southern of North China, Ministry of Agriculture of the People’s Republic of China, Zhengzhou 450002, P. R. China; Institute of Plant Protection, Henan Academy of Agricultural Sciences, Key Laboratory of Crop Pest Control of Henan Province, Key Laboratory of Crop Integrated Pest Management of the Southern of North China, Ministry of Agriculture of the People’s Republic of China, Zhengzhou 450002, P. R. China; Institute of Plant Protection, Henan Academy of Agricultural Sciences, Key Laboratory of Crop Pest Control of Henan Province, Key Laboratory of Crop Integrated Pest Management of the Southern of North China, Ministry of Agriculture of the People’s Republic of China, Zhengzhou 450002, P. R. China; Institute of Plant Protection, Henan Academy of Agricultural Sciences, Key Laboratory of Crop Pest Control of Henan Province, Key Laboratory of Crop Integrated Pest Management of the Southern of North China, Ministry of Agriculture of the People’s Republic of China, Zhengzhou 450002, P. R. China; Institute of Plant Protection, Henan Academy of Agricultural Sciences, Key Laboratory of Crop Pest Control of Henan Province, Key Laboratory of Crop Integrated Pest Management of the Southern of North China, Ministry of Agriculture of the People’s Republic of China, Zhengzhou 450002, P. R. China; Institute of Plant Protection, Henan Academy of Agricultural Sciences, Key Laboratory of Crop Pest Control of Henan Province, Key Laboratory of Crop Integrated Pest Management of the Southern of North China, Ministry of Agriculture of the People’s Republic of China, Zhengzhou 450002, P. R. China

**Keywords:** chromosome-level genome, Hi-C, *Sitodiplosis mosellana*, comparative genomics, detoxification

## Abstract

The orange wheat blossom midge *Sitodiplosis mosellana* Géhin (Diptera: Cecidomyiidae), an economically important pest, has caused serious yield losses in most wheat-growing areas worldwide in the past half-century. A high-quality chromosome-level genome for *S*. *mosellana* was assembled using PacBio long read, Illumina short read, and Hi-C sequencing technologies. The final genome assembly was 180.69 Mb, with contig and scaffold N50 sizes of 998.71 kb and 44.56 Mb, respectively. Hi-C scaffolding reliably anchored 4 pseudochromosomes, accounting for 99.67% of the assembled genome. In total, 12,269 protein-coding genes were predicted, of which 91% were functionally annotated. Phylogenetic analysis indicated that *S*. *mosellana* and its close relative, the swede midge *Contarinia nasturtii*, diverged about 32.7 MYA. The *S*. *mosellana* genome showed high chromosomal synteny with the genome of *Drosophila melanogaster* and *Anopheles gambiae*. The key gene families involved in the detoxification of plant secondary chemistry were analyzed. The high-quality *S*. *mosellana* genome data will provide an invaluable resource for research in a broad range of areas, including the biology, ecology, genetics, and evolution of midges, as well as insect–plant interactions and coevolution.

## Introduction

Gall midges (family Cecidomyiidae) constitute one of the largest families of Diptera, with 6,651 known species and 832 genera (Gagné and Jaschhof 2021). About 75% of Cecidomyiinae species are herbivorous, and many of them induce galls in a great diversity of plants throughout the world (Yukawa and Rohfritsch 2005; Gagné and Jaschhof 2017; Dorchin *et al.* 2019). Meanwhile, most herbivorous gall midges are host specific, developing in one or a few closely related host plants, and many genera and even tribes have specialized and diversified on specific plant families (Gagné and Jaschhof 2017; Dorchin *et al.* 2019). These provide fascinating material for ecological and evolutionary studies.

The orange wheat blossom midge (OWBM), *Sitodiplosis mosellana* Géhin (Diptera: Cecidomyiidae), is an economically important pest and has caused serious yield losses in most wheat-growing areas worldwide (Berzonsky *et al.* 2002; Thomas *et al.* 2005; Bruce *et al.* 2007; Gaafar *et al.* 2011; Gong *et al.* 2013; Jacquemin *et al.* 2014). Larvae feed on young kernels, causing kernel damage, poor seed quality, and lower yield. Moderate invasion by *S*. *mosellana* led to a yield loss of 10–30% in China. The reduction could be as much as 30–60% when severe damage occurs (Duan *et al.* 2013). During the long course of coevolution, insects and host plants have formed intimate relationships, particularly for parasitic insect species. Insect attacks on plants cause extensive changes in gene expression in host plants. In turn, plant defense reactions to insects may cause significant changes in gene expression in insects (Mittapalli *et al.* 2005). Like other gall midges, wheat midge larvae are thought to inject saliva into developing wheat seeds, resulting in shriveled wheat kernels (Lamb *et al.* 2000). Meanwhile, adult oviposition must coincide with wheat heading, since the susceptibility of plants to wheat midge damage declines significantly after anthesis begins (Elliott and Mann 1996; Wu *et al.* 2015). The gene families involved in xenobiotic detoxification will be key in facilitating the successful exploitation of wheat.

Recent studies have focused on the mechanisms of diapause (Li *et al.* 2012), chemical communication (Gong *et al.* 2013; Cheng *et al.* 2020), long-distance migration (Miao *et al.* 2013), evaluation of midge resistance (Hao *et al.* 2019; Zhang *et al.* 2020), biological control (Chavalle *et al.* 2015; Thompson and Reddy 2016; Olfert *et al.* 2020), and wheat midge–wheat interactions (Al-jbory *et al.* 2018). Because of its agricultural importance as the major pest of wheat worldwide, more knowledge about *S*. *mosellana* and its interaction with its host at the molecular level would be useful and benefit from comprehensive genomic analysis. A genome of *S*. *mosellana* was released by Agriculture and Agri-food Canada (NCBI: GCA_009176505.1). However, the genome was fragmented without anchoring on chromosomes. The quality of genome assemblies is the foundation of understanding these biological features; therefore, a more accurate *S*. *mosellana* genome assembly is needed.

Here, we reported a high-quality chromosome-level genome assembly for *S*. *mosellana* using a combination of Illumina, PacBio, and Hi-C technologies. The assembly had high completeness, providing an excellent genomic resource for subsequent research. Moreover, we described cytochrome P450 monooxygenase (P450) and glutathione S-transferase (GST) in *S*. *mosellana*. Our findings provide a valuable genomic resource for molecular and gene family evolutionary studies in midges (e.g. gall-forming evolution), as well as insect–host adaptive evolution, identifying genetic modifications that contribute to its insect–plant lifestyle.

## Materials and methods

### Insects

For genome sequencing, about 50 individuals of *S*. *mosellana* larvae were collected from the wheat ear in Zhumadian city, Henan province, China, in May 2019. At this time, the midge remained a mature third instar and no longer feeds. Prior to DNA preparation, *S*. *mosellana* larvae were starved for 15 days in pure water, and the water was replaced every 2 days to reduce the probability of contamination of the host.

### Genome sequencing and assembly

High-quality DNA extracted from the larvae was used for library preparation and high-throughput sequencing. Short-insert (350 bp) paired-end libraries were prepared according to the Illumina protocol and sequenced on the Illumina NovaSeq 6000 (Illumina, Inc.) with a paired-end 150 (PE150) read layout and yield a total 28.67 Gb of sequencing data. This content corresponded to 171.49-fold genome coverage. Whole-genome sequencing was performed using the PacBio Sequel sequencer (Pacific Biosciences). The 20-kb single-molecule real-time sequencing bell libraries were constructed using the standard protocol. The PacBio Sequel platform generated 17.82 Gb of sequencing data, representing 106.59-fold coverage depth.

All raw reads from the PacBio platform were subjected to error correction according to the rate of insertions, deletions, and sequencing errors between the base pairs to obtain preassembled reads ‘daligner’ (Myers 2014). Then, the preassembled reads were assembled by the consensus algorithm called Overlap-Layout-Consensus to obtain contigs using FALCON (Chin *et al.* 2016). Overlapping reads and raw subreads were processed to generate consensus sequences, and the error correction of the assembly was polished using the consensus-calling algorithm Quiver (Chin *et al.* 2013). The paired-end clean reads from the Illumina platform were further corrected for any remaining errors using Pilon (Walker *et al.* 2014). Finally, the Purge Haplotigs pipeline was run to produce an improved, deduplicated assembly (Roach *et al.* 2018).

To assist chromosome-level assembly, we used the high-throughput chromosome conformation capture (Hi-C) technique to capture genome-wide chromatin interactions (Belaghzal *et al.* 2017). For Hi-C sequencing, the chromosomal structure was fixed by formaldehyde crosslinking, and then the MboI enzyme was used to shear DNA. A Hi-C library with 350 bp insert size was constructed, which was sequenced on an Illumina NovaSeq 6000 according to the manufacturer’s instructions. The Hi-C library generated 30.35 Gb sequencing data, which corresponds to approximately 181.54-fold coverage of the *S.* *mosellana* genome. We then used the pruning, partition, rescue, optimization, and building of the ALLHiC pipeline (Dudchenko *et al.* 2017; Zhang *et al.* 2019) to construct the chromosome-level scaffolds of the *S*. *mosellana* genome.

### Transcriptome sequencing

To assist in genome annotation, different transcriptome profiles were generated from different sexes (male and female adults) and various developmental stages (diapause larvae, nondiapause larvae, pupae) with 2 replications (NCBI SRA: SRX 12027781–SRX 12027792). In total, 12 cDNA libraries were prepared. A total amount of 3-µg RNA per sample was used as input material for RNA sample preparation. Sequencing libraries were generated using the NEBNext Ultra RNA Library Prep Kit for Illumina (NEB, USA) following the manufacturer’s recommendations. Libraries were sequenced on an Illumina NovaSeq 6000 platform, and paired-end reads were generated.

Transcriptome reads assemblies were generated with Trinity (v2.1.1) for the genome annotation. To optimize the genome annotation, the RNA-Seq reads from different tissues, which were aligned to genome fasta using Hisat (v2.0.4)/TopHat (v2.0.13) with default parameters to identify exons region and splice positions. The alignment results were then used as input for Stringtie (v1.3.3)/Cufflinks (v2.1.1) with default parameters for genome-based transcript assembly. The nonredundant reference gene set was generated by merging genes predicted by 3 methods with EvidenceModeler (EVM, v1.1.1) (Haas *et al.* 2008) using program to assemble spliced alignment (PASA) (Haas *et al.* 2003) terminal exon support and including masked transposable elements (TEs) as input into gene prediction. Individual families of interest were selected for further manual curation by relevant experts.

### Assessment of completeness of the assembly and gene set

To assess the accuracy of the assembled *S*. *mosellana* genome, a small fragment library was selected for comparison of the assembled genome using BWA software (http://bio-bwa.sourceforge.net/) (Li and Durbin 2009). To assess the completeness of the assembly and gene annotation, we performed analysis with Benchmarking Universal Single-Copy Orthologs (BUSCO, version 3.0) (http://busco.ezlab.org/) with default parameters (Waterhouse *et al.* 2018).

### Genome annotation

Repeat sequences and TEs were identified using both homology-based and de novo prediction methods. For de novo predictions, LTR_FINDER (v1.0.6) (http://tlife.fudan.edu.cn/ltr_finder/), RepeatScout (v1.0.5) (http://www.repeatmasker.org/), and RepeatModeler (v2.0.1) (http://www.repeatmasker.org/RepeatModeler.html) were used to construct a de novo repeat library with default parameters, then all repeat sequences with lengths >100 bp and gap “*N*” less than 5% constituted the raw TE library. Prediction of tandem repeats were also searched using Tandem Repeats Finder (http://tandem.bu.edu/trf/trf.html) with the following parameters: Match= 2, Mismatch= 7, Delta= 7, PM= 80, PI= 10, Minscore= 50, MaxPeriod= 2,000. For homology-based predictions, RepeatMasker (v4.1.0) (http://repeatmasker.org/) was used with Repbase library (Bao *et al.* 2015). In addition, we used RepeatProteinMask (http://www.repeatmasker.org/) with the default parameters to identify repeat sequences at the protein level.

The tRNAs were predicted using the tRNAscan-SE program, whereas ribosomal RNAs (rRNAs) were predicted using BLASTN. Other ncRNAs, including microRNAs (miRNAs) and small nuclear RNAs (snRNAs), were identified by searching against the Rfam database with default parameters using the INFERNAL software.

For gene structure prediction, homology-based prediction, ab initio prediction, and transcriptome-based prediction were combined to predict protein-coding genes in the *S. mosellana* genome based on the repeat masked genome. For the former, protein sequences from *Mayetiola destructor*, *Belgica antarctica*, *Drosophila melanogaster*, *Aedes aegypti*, *Culex quinquefasciatus*, *Anopheles gambiae*, *Bactrocera dorsalis* were aligned to the *S. mosellana* genome using TBLASTN (*E*-value ≤ 1E-05). Then Genewise (version 2.4.1) (Birney *et al.* 2004) was used for further precise alignment and gene structure prediction. For the ab initio-based method, Augustus (v3.2.3) (Stanke *et al.* 2006), GlimmerHMM (v3.0.4) (Majoros *et al.* 2004), SNAP (v2013.11.29) (Leskovec and Sosič 2016), Geneid (v1.4) (Parra *et al.* 2000), and Genscan (v1.0) (Burge and Karlin 1997) were employed as engines to predict gene models. For RNA-seq-based prediction, transcriptome data from 12 samples were aligned to the assembled genome sequence using Hisat2 (v2.0.4) (Kim *et al.* 2015). The alignment results were then used as input for Stringtie (v1.3.3) (Pertea *et al.* 2015) with default parameters for genome-based transcript assembly. The gene prediction results derived from 3 strategies were merged using EVM (v1.1.1) (Haas *et al.* 2008) to generate a consensus gene set. Finally, PASA (Haas *et al.* 2003) was used to acquire the final gene structures after adjusting the gene models generated from EVM with the transcripts assembled by Trinity (v2.1.1) (Grabherr *et al.* 2011).

Gene functional annotation was performed based on homologue searches and the best match to the databases of Kyoto encyclopedia of genes and genomes (KEGG: http://www.genome.jp/kegg/) (Kanehisa and Goto 2000), SwissProt (http://www.uniprot.org/) (Bairoch and Apweiler 2000), nonredundant proteins (NR: http://www.ncbi.nlm.nih.gov/protein), and Pfam (http://pfam.xfam.org/) (El-Gebali *et al.* 2019). The Gene Ontology (GO: http://www.geneontology.org/) (Ashburner *et al*. 2000) analysis was executed through InterPro (https://www.ebi.ac.uk/interpro/) (Mulder and Apweiler 2007) to identify protein domains. The information from different sources of functional annotation was combined for each gene in the final integration.

### Comparative genomics and phylogenetic reconstruction

Protein-coding genes from another 11 species of Diptera, as well as 1 species of Coleoptera, 1 species of Lepidoptera, 1 species of Hymenoptera, and 1 common water flea (*Daphnia pulex*), in the order of Anomopoda, were obtained from the NCBI genomes database for comparative analysis. *D*. *pulex* was used as an outgroup. Orthologues were identified using OrthoMCL (Li *et al.* 2003). Orthologous groups that contained only 1 gene for each species were represented by the gene encoding the longest protein sequence. Genes encoding protein sequences shorter than 50 amino acids were filtered out to exclude putative fragmented genes. All-vs-all BLASTP was applied to identify similarities among the filtered protein sequences in these species with an *E*-value cut-off of 1e^−5^. Muscle (Edgar 2004) with default parameters was used to generate a multiple sequence alignment of the protein sequences in each single copy family. The alignments of each family were then concatenated to form a super alignment that was used for phylogenetic tree reconstruction using RAxML maximum-likelihood methods with the model LG + F + I + G4 with “-m PROTGAMMAAUTO -p 12345 -x 12345 -# 100 -f ad” (Guindon *et al.* 2010; Yang and Rannala 2012; Stamatakis 2014). Statistical support was obtained with 1,000 bootstrap replicates. The species divergence time was estimated using MCMCTree in the PAML version 4.9 package (Yang 2007) with default parameters. The calibration information for MCMCTree was extracted based on the TimeTree database (http://www.time.org/).

### Gene family expansion and contraction

To further explore gene family changes under natural selection, the expansion and contraction of gene families were identified using the likelihood model originally implemented in the software package CAFE version 4.2 (De Bie *et al.* 2006) with the following parameters: “-p 0.05 -t 4 -r 10000.” Gene families only present in *S*. *mosellana* but absent in other species were considered group specific. We used Fisher's exact test to identify overrepresented GO and KEGG pathways among the expanded and contracted genes, followed by a false discovery rate correction (FDR < 0.05).

### Synteny analysis

Whole-genome sequence alignments between *S*. *mosellana*, *D*. *melanogaster*, and *A*. *gambiae* were detected and plotted using mcscan (https://github.com/tanghaibao/jcvi/wiki/MCscan-; Python-version) with default parameters (Tang *et al.* 2008). The chromosome-level genome assembly of *D*. *melanogaster* (Adams *et al.* 2000) was downloaded at https://www.ncbi.nlm.nih.gov/assembly/GCF_000001215.4#/def. The chromosome-level genome assembly of *A*. *gambiae* (Holt *et al.* 2002) was downloaded at https://www.ncbi.nlm.nih.gov/assembly/GCF_000005575.2#/def.

### Identification and phylogenetic analysis of detoxification genes in the *S*. *mosellana* genome

To uncover the potential detoxification genes, the *S. mosellana* predicted protein sequences were used as queries in the blast searches to the NCBI Nr database with 1 × 10^−5^*E*-value threshold. In this study, the *S*. *mosellana*-predicted protein sequences were classified into Sm-P450 and Sm-GST sequences, as the one of the top 10 hits of them was annotated by cytochrome P450 and GST, respectively.

The protein sequences of detoxification genes were retrieved in genomes of *D*. *melanogaster* (assembly Release 6 plus ISO1 MT) and *Mayetiola destructor* (Zhao *et al.* 2015) to uncover the phylogenetic positions of *S*. *mosellana*-related genes. The sequences were aligned with MUSCLE, as implemented in MEGA 7.0 (Kumar *et al.* 2016). The phylogenetic analysis was performed using IQ-TREE 1.6.6 (Nguyen *et al.* 2015). The substitution model was selected in Mod-elFinder (Kalyaanamoorthy *et al.* 2017) with the Bayesian information criterion. The ultrafast bootstraps were resampled with 5,000 runs to assess the support for each node. The phylogenetic trees were visualized using the ggtree R package (Yu *et al.* 2017).

## Results and discussion

### Features of the assembled genome

Based on the Illumina reads, the genome size of *S*. *mosellana* was estimated to be 167.18 Mb, based on 17 K-mer analysis (Supplementary Fig. 1 and Supplementary Table 2). The heterozygosity rate was 1.94%. The high heterozygosity of the *S*. *mosellana* genome might be caused by pooling the DNA of multiple individuals for short-read sequencing. Our study demonstrates that the current methods are appropriate for high-quality de novo assembly of the genome of small highly heterozygous organism sequencing projects (Lian *et al.* 2019; Guo *et al.* 2020; Ye *et al.* 2020).

**Fig. 1.**
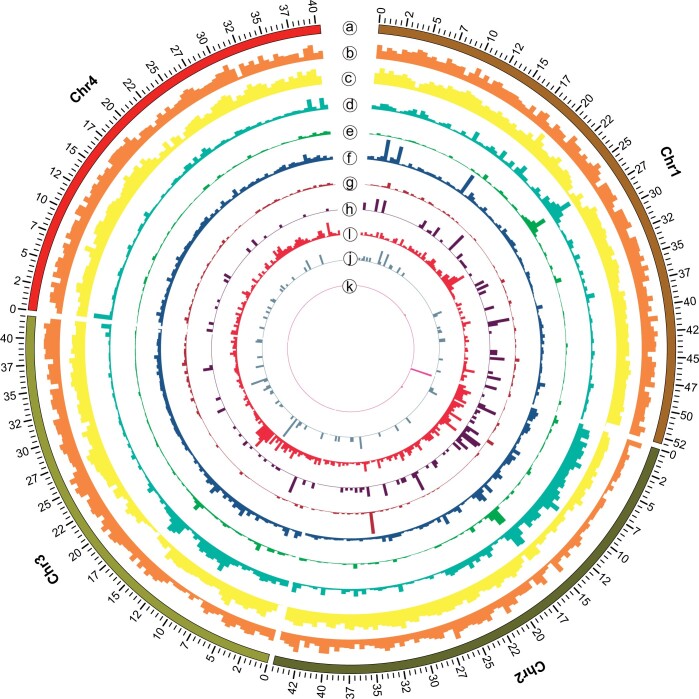
The genome characteristics of OWBM, *S. mosellana*. Circos plot showing the genomic features. Units on the circumference are megabase values of pseudomolecules. From outermost to innermost circles: Track a: 4 chromosomes of the genome; Track b: gene distribution on each chromosomes; Track c: GC content distribution on each chromosomes; Track d: LTR distribution on each chromosomes; Track e: LINE distribution on each chromosomes; Track f: DNA distribution on each chromosomes; Track g: SINE distribution on each chromosomes; Track h: tRNA located on chromosomes; Track i: miRNA located on chromosomes; Track j: snRNA located on chromosomes; Track k: rRNA located on chromosomes.

The genome of the OWBM *S*. *mosellana*, sequenced using both PacBio and Illumina HiSeq 2000 platforms, generated 17.8 Gb PacBio long reads and 28.7 Gb Illumina short reads, with 278.11× genome coverage. We obtained a reference *S*. *mosellana* genome of 180.66 Mb with a contig N50 of 988.71 kb. The GC content of the *S*. *mosellana* genome was 36.4% (Supplementary Fig. 2 and Supplementary Table 2). Hi-C technology was then used to improve genome assembly to the chromosomal level. A total of 30.35 Gb clean reads were generated, accounting for 181.54-fold coverage. The Hi-C scaffolding was able to anchor and order all 25 scaffolds into 4 chromosomes, with more than 91.74% of assembled bases located on the chromosomes (Fig. 1 and Supplementary Fig. 3 and Supplementary Tables 3 and 4). The length of the largest chromosome was 53.05 Mb, while the smallest chromosome was 40.43 Mb (Supplementary Table 4). The final genome assembly was approximately 180.69 Mb, with scaffold and contig N50 sizes of 44.56 Mb and 998.71 kb, respectively (Table 1). The assembled genome size was slightly higher than that obtained by K-mer estimation (167.18 Mb; Supplementary Fig. 1) and was similar to *C*. *nasturtii* (Mori *et al.* 2021) and *M. destructor* (Zhao *et al.* 2015). For the *S*. *mosellana* genome released by Agriculture and Agri-food Canada, the assembly size was 208 Mb and the scaffold N50 was 5.13 Mb, which was not a chromosome-level assembly (Table 1). The scaffold N50 of this genome was 44.56 Mb, making it a high quality, and potentially the best quality, *S*. *mosellana* genome available to date. These results showed that the genome reported in the current study had a high level of continuity and completeness.

**Table 1.** Comparison of *S. mosellana* genome assemblies from this and a previous study.

Assembly	ASM2101890v1 (this study)	AAFC_SMos_1.0 (from Agriculture and Agri-food Canada)
Bioproject	PRJNA720212	PRJNA563698
DNA resource	Third-instar larvae	Single pupa
Assembly approach	Falcon	Supernova
Sequencing platform	NovaSeq/PacBio	Illumina HiSeq
Assembly level	Chromosomes	Scaffolds
Number of contigs	381	11,287
Contig N50 (bp)	988,708	62,752
Number of Scaffolds	25	7,269
Scaffold N50 (bp)	44,562,869	5,125,045
Total gap length (bp)	35,600	13,573,270
Total sequence length	180,693,642	208,800,104
Ungapped bases (bp)	180,658,042	195,226,834

For quality evaluation of the genome assembly, according to BWA software (http://bio-bwa.sourceforge.net/), a total of 91.7% of the short reads were uniquely mapped to the genome assembly and the coverage rate was 99.8%, indicating that the assembled genome was high quality (Supplementary Table 5). A BUSCO assessment showed that 93.1% of BUSCO genes were successfully detected, of which 90.7% were single copy and 2.0% were duplicated (Table 2). Compared to the Insecta databases, the results showed a high-quality assembly of *S. mosellana* above 90% of conserved genes of the database. The results of these 2 evaluations indicated that the genome assembly had a high level of completeness and was suitable for subsequent analysis.

**Table 2.** Statistics of the completeness of the assembled *S. mosellana* genome by BUSCO.

Type	BUSCO groups	Percentage (%)
Complete BUSCOs	907	92.7
Complete and single-copy BUSCOs	887	90.7
Complete duplicated BUSCOs	20	2.0
Fragmented BUSCOs	4	0.4
Missing BUSCOs	67	6.9
Total BUSCO groups searched	978	100

In addition, to measure genome-wide sequencing bias, the GC content and average depth of the assembled genome were calculated and mapped using 10-kb nonoverlapping slide windows. The density points (red scatter plot) only concentrated within the 30–40% range, with the average GC content of 36.4% (Supplementary Fig. 2).

### Genome annotation

Repetitive elements, including TEs, are a major sequence component of eukaryote genomes (Petersen *et al.* 2019). RepeatMasker (http://www.repeatmasker.org/) software and Repbase (http://www.girinst.org/repbase) database annotated the repeat sequences. The results of repeat prediction showed that the *S*. *mosellana* genome contains 21.55% repeat sequences. Repetitive sequence statistics and classification results are shown in Supplementary Table 6. Short interspersed nuclear elements (SINEs), long interspersed nuclear elements (LINEs), long terminal repeats (LTRs), and DNA elements accounted for 0.02%, 0.86%, 13.24%, and 1.67% of the whole genome, respectively, and 6.57% of repeat sequences were annotated as unclassified (Fig. 1 and Supplementary Fig. 4 and Supplementary Table 7). The TEs represented 21.09% of the whole *S. mosellana* genome. Similarly, TEs occupied approximately 16% of the *M. destructor* genome (Ben Amara *et al.* 2021) and 13.9% of the *C. nasturtii* genome (Mori *et al.* 2021). TE content varies greatly among the insects and differs even between species belonging the same order. In the Diptera species, TE content ranges from less than 1% in *Belgica Antarctica* to around 50% in *Aedes aegypti*. Similar proportions were estimated in other Dipteran genomes like the Drosophilidae species whose TE content varies between 3% and 25% (Clark and Pachter 2007).

A total of 224 tRNAs were predicted by tRNAscan-SE (http://lowelab.ucsc.edu/tRNAscan-SE/) (Chan and Lowe 2019). Using Blast, 18 rRNAs were identified. Using infernal software (http://infernal.janelia.org/) (Nawrocki and Eddy 2013), we also identified 21 scaRNA, 80 snRNAs, 1,406 miRNAs, and 59 other ncRNAs (Fig. 1 and Supplementary Table 8).

Gene structure prediction was performed, and 12,269 protein-coding genes were predicted, with a mean of 1,520.74 bp of coding sequence (CDS) and 5.18 exons per gene (Supplementary Table 9 and Supplementary Fig. 5). The transcript lengths of genes, CDSs, exons, and introns of *S*. *mosellana* are comparable to those of the genomes used for homology-based prediction (Supplementary Table 10 and Supplementary Fig. 5). The genome of *S.* *mosellana* is larger than the genome reported for the *Belgica antarctica* (99 Mb) (Kelley *et al.* 2014). *S. mosellana* genes tend to have much longer introns than do those of *B. antarctica*. Similarly, the genome of *Aedes aegypti*, *Culex quinquefasciatus*, and *A. gambiae* were larger than the genome of *S. mosellana*. The mosquito genes had longer introns than those of *S. mosellana*. The intron size comparison showed that a reduction in intron length also contributed to the reduced size of this genome.

Of all predicted protein-coding genes, 88.6% (10,869) had BLAST hits in the NCBI nonredundant database. Furthermore, 58.7% (7,201) were assigned GO terms, and 75.4% (9,245) were mapped to at least 1 KEGG pathway (Table 3 and Supplementary Fig. 6).

**Table 3.** Statistics of gene function annotation of *S. mosellana.*

Database	Number	Percentage (%)
Total	12,269	–
Swissprot	9,292	75.70
Nr	10,869	88.60
KEGG	9,245	75.40
InterPro	10,235	83.40
GO	7,201	58.70
Pfam	9,121	74.30
Annotated	11,169	91.00
Unannotated	1,100	9.00

### Evolutionary analysis

**Fig. 2.**
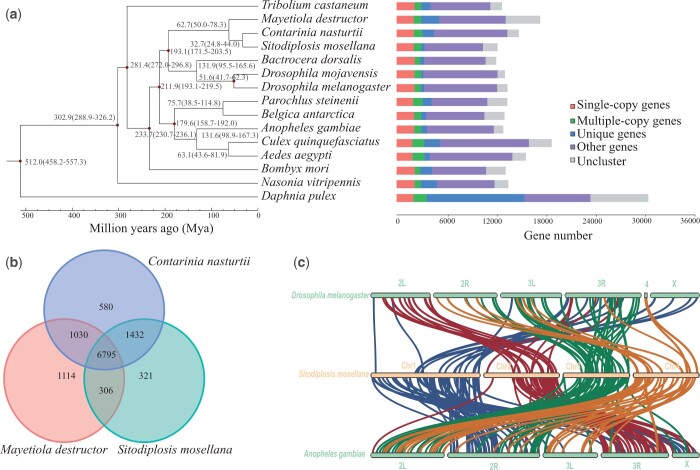
Phylogenetic tree, gene orthology, and synteny blocks. a) The phylogenetic tree was constructed based on 1,024 single-copy gene families with 14 insects and 1 noninsect species, using RAxML maximum-likelihood methods. Bootstrap values are 100 in all nodes based on 100 replicates. The numbers near each node correspond to the estimated divergence time of these species. The colored bars to the right are subdivided to represent different types of orthology. “Single-copy genes” indicates single copy orhologous genes in common gene families; “Multiple-copy genes” indicates mutiple copy orthologous genes in common gene families; “Unique genes” indicates genes from unique gene family from each species; “Other genes” indicates genes that do not belong to any above-mentioned ortholog categories; “Uncluster” indicates genes that do not cluster to any families. b) Venn diagram of the orthologous gene families from 3 gall midges: *S. mosellana*, *C. nasturtii*, and *M. destructor*. c) Synteny blocks between *S. mosellana*, *D. melanogaster*, and *A. gambiae*.

Dipteran diversity was traditionally partitioned into 2 principal suborders: the Nematocera and the Brachycera (Wiegmann *et al.* 2011). The gall midges, along with marsh flies, gnats, and other midges, made up the nematoceran infraorder, Bibionomorpha. Protein sequences from the 1,024 single-copy gene families were used for phylogenetic tree reconstruction, and the estimation of divergence time was performed (Fig. 2a) with the MCMC tree program implemented in the PAML. The results showed that *S*. *mosellana* and 10 other flies were clustered together (Fig. 2a). Our analysis showed that the OWBM *S*. *mosellana*, Swede midge *C*. *nasturtii*, and Hessian fly *M*. *destructor* formed a sister lineage to Cecidomyiidae, while *D*. *melanogaster*, *D*. *mojavensis*, and *B*. *dorsalis* were in another sister lineage. Therefore, the placement of *S*. *mosellana*, *C*. *nasturtii*, and *M. destructor* with the Drosophilids (Brachycera) confirmed the Nematocera to be a paraphyletic group, consistent with previous analyses placing the Bibionomorpha as a sister group to the Brachycera (Wiegmann *et al.* 2011; Zhao *et al.* 2015). The genes used for gene family clustering in each species are shown in Supplementary Table 11. In total, 1,024 single-copy gene families are common to all 15 species. The distributions of single-copy orthologs, multiple-copy orthologs, genes unique to *S*. *mosellana*, and other orthologs in different species are shown in Fig. 2a and Supplementary Table 12. Overall, 321 gene families (564 genes included) were unique to *S*. *mosellana*.

The family Cecidomyiinae usually was divided into 2 supertribes: the Lasiopteridi and the Cecidomyiidi. The genera Mayetiola was in the former, while Contarinia, and Sitodiplosis are in the latter (Hall *et al.* 2012; Dorchin *et al.* 2019). A total of 6,795 homologous gene families were shared by the 3 species. *S*. *mosellana* shared 8,191 gene families with *C*. *nasturtii* and more than 7,101 with *M*. *destructor* (Fig. 2b), which showed more homology between *S*. *mosellana* and *C*. *nasturtii*.

Estimated divergence times of *S*. *mosellana* and other species (calculated using MCMCTREE) suggest that *S*. *mosellana* diverged from the common ancestor of *C*. *nasturtii* 32.7 MYA, and from the ancestor of *M*. *destructor* 62.7 MYA. Thus, the divergence of *S*. *mosellana* postdated that of *M*. *destructor*, a plant parasitic gall midge and a pest of wheat (*Triticum* spp.). The split of the Neodiptera lineage from other Diptera clusters was inferred to be around 211.9 MYA. All 11 Diptera insects diverged from the sister lineage *B*. *mori* about 233.7 MYA (Fig. 2a). The fly phylogenetic relationships were consistent with previous studies (Wiegmann *et al.* 2011; Vicoso and Bachtrog 2015).

### Expansion and contraction of gene families in *S*. *mosellana*

**Fig. 3.**
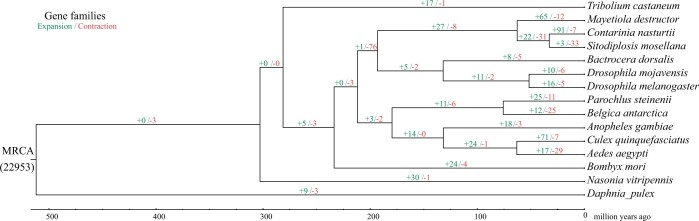
Gene family evolution between genomes of *S. mosellana* and 14 other arthropod species. Left number indicates gene family expansions and right number indicates gene family contractions. The length of branch indicate the divergence time. MRCA: most recent common ancestor.

Of the 22,953 gene families in the most recent common ancestor (MRCA) of all 15 species, 3 were expanded and 33 were contracted in *S*. *mosellana* compared with gene families of the common ancestor of *S*. *mosellana* and *C*. *nasturtii* (Fig. 3). In contrast, *C*. *nasturtii* had 91 expanded and 7 contracted gene families. The common ancestor of Cecidomyiidae species showed 27 expanded and 8 contracted gene families compared to that of the common ancestor of Drosophilidae species and Tephritidae.

GO enrichment analysis reveal that the *S. mosellana*-contracted gene families are enriched in carbohydrate metabolic process (GO:0005975, 4 genes, *P* = 0.002629, Adjusted *P*-value), oxidation–reduction process (GO:0055114, 5 genes, *P* = 0.005992), sensory perception of smell (GO:0007608, 2 genes, *P* = 0.006189) (Supplementary Table 13).

As *S. mosellana* adults did not feed and larvae had no capability for host selection, a reduced role for sensory perception was consistent with the general loss of chemoreceptors, the same way as in *M. destructor* (Zhao *et al.*, 2015).

### Chromosome synteny

Synteny referred to genes that reside on the same chromosome. Conserved synteny indicated that homologous genes were syntenic between species, regardless of gene

order (Ehrlich *et al.* 1997). Syntenic relationships between *S*. *mosellana*, *D*. *melanogaster*, and *A*. *gambiae* showed a high level of collinearity among the 3 chromosome-level genomes, and a relatively low frequency of fragment rearrangements was observed (Fig. 2c). We defined a syntenic block as including at least 3 orthologous genes. In total, 48 syntenic blocks were found between *S*. *mosellana* and *D*. *melanogaster*, and the gene number in these blocks ranged from 4 to 12, with a mean of 5.31. Eighty-one blocks were found between *S*. *mosellana* and *A*. *gambiae*, with the same gene number range of 4–15 and a mean of 6.20. The most conserved pairs of chromosomal arms were SmChr2/Dm2L and SmChr2/Ag3R, with 75% and 80% of synteny blocks in SmChr2 mapping to Dm2L and Ag3R, respectively. The remaining blocks represented exchanges with other arms. Other relationships were 70% and 46% of synteny blocks in SmChr1 mapping to Dm3R and Ag 2R, respectively. In our analysis, *S*. *mosellana* showed slightly higher synteny with *A*. *gambiae* than *D*. *melanogaster*, despite the closer phylogenetic relationship of *S*. *mosellana* and *D*. *melanogaster*.

Gene families were commonly found in genomes and were thought to evolve by gene duplication and neofunctionalization. The Osiris gene family was a large conserved family first described in *D. melanogaster* (Dorer *et al.* 2003). Twenty-three Osiris genes were originally found in the *D. melanogaster* genome, with 20 of them located on chromosome 3R in a cluster. The Osiris gene family was also present in the mosquito *A. gambia* genome (Dorer *et al.* 2003) and *S. mosellana* genome. The families maintained a remarkable degree of synteny displays remarkable synteny and sequence conservation with the *Drosophila* cluster (Shah *et al.* 2012).

### Evolution of detoxification gene families in *S*. *mosellana*

Herbivorous insects have developed detoxification enzymes to metabolize otherwise deleterious plant secondary metabolites (Ramsey 2010; Simon *et al.* 2015). As a strict specialist, *S*. *mosellana* likely had adaptations that allowed it to detoxify these chemicals (Smith *et al.* 2004, 2007). The vast array of GST and CYP450 genes in insects represents the largest repertoire of detoxification enzyme genes known (Hazzouri *et al.* 2020). There were 4 large clades of insect P450 genes: the CYP2 clade, the CYP3 clade, the CYP4 clade, and the mitochondrial clade (Feyereisen 2006). With homology searching, 95 P450 genes were annotated and grouped into the 4 major clades (Supplementary Fig. 7). CYP3 ranked as the largest clade, consisting of 51 members, and strong gene expansion was observed (36 for *D*. *melanogaster* and 38 for *M*. *destructor*). The CYP4 clade included 23 P450 members. The remainder belonged to the mitochondrial (10 ones) and CYP2 (11 ones) clades. CYP6 and CYP313 were the most expanded gene families, with a more specific expansion of subfamilies CYP6D (25 genes) (Supplementary Fig. 7). Other examples of several such blooms in a diversity of species are the 17 CYP6AS genes in honeybee (Claudianos *et al.* 2006), 12 CYP6A genes in the fruit fly (Tijet *et al.* 2001), and 13 CYP6BQ genes in *T*. *castaneum* (Zhu *et al.* 2013). Although few of the CYP6 enzymes have been characterized and in many (but not all) studies, they are shown to metabolize xenobiotics and plant natural compounds (Li *et al.* 2002; Feyereisen 2012; Edi *et al.* 2014). Greater expression levels of P450 genes were found in *M*. *destructor* and *Aphis glycine* feeding on resistant plants (Bansal *et al.* 2014; Chen *et al.* 2016). In fact, the induction of xenobiotic response genes by plant secondary metabolite exposure was thought to be the first step leading to eventual detoxification and virulence adaptation (Bansal *et al.* 2014).

Another group of detoxification enzymes was GSTs. GSTs are involved in many cellular physiological activities, such as detoxification of endogenous and xenobiotic compounds, intracellular transport, biosynthesis of hormones, and protection against oxidative stress (Enayati *et al.* 2005; Shi *et al.* 2012). In *S*. *mosellana*, 26 GST genes were identified. GST genes were grouped into 5 GST classes, with 5 Delta GSTs, 14 Epsilon GSTs, 1 Omega GST, 5 Theta GSTs, and 1 Sigma GST. Genes belonging to the Zeta class were not found. Contractions of GST genes were derived from Delta classes (Supplementary Fig. 8). The similar numbers of GST genes in *S*. *mosellana* and *M*. *destructor* (22 genes) were in contrast with the significantly higher number of GST genes in *D*. *melanogaster* (36 genes). This might correspond to midges’ narrow host range. *S*. *mosellana* and *M*. *destructor* had similar diets (consisting mainly of wheat) and habitats (primarily wheat dominated). The great number of orthologous GST gene groups in *S*. *mosellana* and *M*. *destructor* species suggested that the radiation event or independent expansion of the GST gene family in these species may have occurred relatively recently, and this was consistent with previous studies in 3 planthoppers (Zhou *et al.* 2013). As in *Drosophila* and *Ceratitis capitata*, many of the insect-specific genes of the Delta and Epsilon subclasses are putatively involved in insect responses to environmental conditions, as well as in xenobiotic and insecticide resistance (Enayati *et al.* 2005; Li *et al.* 2007; Papanicolaou *et al.* 2016).

## Conclusion

In summary, we successfully assembled a genome for the wheat pest *S*. *mosellana*, providing the first chromosome-level genome for a species from the family Cecidomyiidae of Diptera insects using Illumina and PacBio sequencing platforms with Hi-C technology. The availability of the genome sequence will facilitate the future evaluation of unique biological characteristics of *S*. *mosellana*, such as olfactory reception, prolonged diapauses, and insect–host interactions.

## Data availability

The raw reads and assembled genome produced in this study were deposited in the National Center for Biotechnology Information (NCBI) with the BioProject accession number PRJNA720212. The whole genome sequence reported in this article was deposited in the Genome Warehouse in the Beijing Institute of Genomics Data Center (https://bigd.big.ac.cn/) under accession number GWHBEIQ00000000.

Supplemental material is available at *G3* online.

## Supplementary Material

jkac161_Supplementary_Data
