# Supplementary material for: A chromosome-level genome assembly of the orange wheat blossom midge, Sitodiplosis mosellana Géhin (Diptera: Cecidomyiidae) provides insights into the evolution of a detoxification system
Source: G3 (Bethesda). 2022 Jun 25;12(8):jkac161. doi: 10.1093/g3journal/jkac161 (PMC9339269; doi:10.1093/g3journal/jkac161)
Supplement: jkac161_Supplementary_Data [file jkac161_supplementary_data.docx]

**Supplemental Information for:**

**A chromosome-level genome assembly of the orange wheat blossom midge, *Sitodiplosis mosellana* Géhin (Diptera: Cecidomyiidae) provides insights into the evolution of a detoxification system**

Zhongjun Gong^1#^, Tong Li^1#^, Jin Miao^1^, Yun Duan^1^, Yueli Jiang^1^, Pei Guo^1^, Huiling Li^1^, Xueqin Wang^1^, Jing Zhang^1^, Yuqing Wu^1*^

^1^Institute of Plant Protection, Henan Academy of Agricultural Sciences, Key Laboratory of Crop Pest Control of Henan Province, Key Laboratory of Crop Integrated Pest Management of the Southern of North China, Ministry of Agriculture of the People’s Republic of China, Zhengzhou, PR China Zhengzhou 450002, P. R. China,

* Correspondence

Yuqing Wu, Institute of Plant Protection, Henan Academy of Agricultural Sciences, Zhengzhou, China.

E-mail: [yuqingwu36@hotmail.com](mailto:yuqingwu36@hotmail.com)

# These authors contributed equally to this work.

**Table of Contents:**

| **Table S1** | Page 3 |
| --- | --- |
| **Table S2** | Page 4 |
| **Table S3** | Page 5 |
| **Table S4** | Page 6 |
| **Table S5** | Page 7 |
| **Table S6** | Page 8 |
| **Table S7** | Page 9 |
| **Table S8** | Page 10 |
| **Table S9** | Page 11 |
| **Table S10** | Page 12 |
| **Table S11** | Page 13 |
| **Table S12** | Page 14 |
| **Table S13** | Page 15 |
| **Figure S1** | Page 16 |
| **Figure S2** | Page 17 |
| **Figure S3** | Page 18 |
| **Figure S4** | Page19 |
| **Figure S5** | Page 20 |
| **Figure S6** | Page 21 |
| **Figure S7** | Page 22 |
| **Figure S8** | Page 23 |
| **Code Availability** | Page 24 |

**Table S1 Estimate the genome size with 17-mer.**

| **Kmer** | **Kmer Depth** | **Kmer Number** | **Genome_size(M)** | **Revised Genome_size(M)** | **Heterozygous_rate(%)** | **Repeat_rate(%)** |
| --- | --- | --- | --- | --- | --- | --- |
| 17 | 37 | 6,366,563,876 | 172.07 | 167.18 | 1.94 | 29.56 |

**Table S2 Statistics of base content of the *S. mosellana* genome**

|  | Number (bp) | % of genome |
| --- | --- | --- |
| A | 57,473,963 | 31.81 |
| T | 57,446,374 | 31.79 |
| C | 32,878,021 | 18.20 |
| G | 32,859,684 | 18.19 |
| N | 35,600 | 0.02 |
| total | 180,693,642 | -- |
| GC | 65,737,705 | 36.39 |

**Table S3 Summary of sequencing data volume for draft genome assembly**

| Pair-end libraries | Insert size | Total data (G) | Read length (bp) | Sequence coverage (X) |
| --- | --- | --- | --- | --- |
| Illumina reads | 350bp | 28.67 | 150 | 171.49 |
| PacBio reads | -- | 17.82 | -- | 106.59 |
| Hi-C | -- | 30.35 | -- | 181.54 |
| Total | -- | 76.84 | -- | 459.62 |

**Table S4 Overview of chromosome length and Cluster numbers on each chromosome of *S. mosellana* assembly**

| Chr_ID | Cluster Number | Chr_Length (bp) |
| --- | --- | --- |
| Chr1 | 74 | 53,047,358 |
| Chr2 | 123 | 44,562,869 |
| Chr3 | 98 | 42,058,288 |
| Chr4 | 65 | 40,427,606 |

**Table S5 Statistics of reads coverage of the *S. mosellana* genome**

|  |  | % of Percentage |
| --- | --- | --- |
| Reads | Mapping rate (%) | 91.74 |
| Genome | Average sequencing depth | 124.93 |
|  | Coverage (%) | 99.80 |
|  | Coverage at least 4X (%) | 99.62 |
|  | Coverage at least 10X (%) | 99.28 |
|  | Coverage at least 20X (%) | 98.61 |
|  |  |  |

**Table S6 Statistics of repeated sequence in *S. mosellana* genome**

| **Type** | **Repeat Size(bp)** | **% of genome** |
| --- | --- | --- |
| Trf | 3,221,798 | 1.78 |
| Repeatmasker | 37,966,242 | 21.01 |
| Proteinmask | 1,963,894 | 1.09 |
| Total | 38,933,351 | 21.55 |

**Table S7 Summary of different types of repeat elements for *S. mosellana***

| Type | Length (bp) | % in Genome |
| --- | --- | --- |
| SINEs | 31,910 | 0.02 |
| LINEs | 1,559,872 | 0.86 |
| LTR elements | 23,932,469 | 13.24 |
| DNA elements | 3,010,140 | 1.67 |
| Unknown | 11,873,236 | 6.57 |
| Total | 38,105,034 | 21.09 |

**Table S8 Statistics of noncoding RNA of *S. mosellana* genome**

|  | Type | Copy number | Average length(bp) | Total length(bp) |
| --- | --- | --- | --- | --- |
| miRNA | | 1,406 | 173.96 | 244,594 |
| tRNA | | 224 | 77.18 | 17,288 |
| rRNA | rRNA | 9 | 193.22 | 1,739 |
|  | 18S | 6 | 197.17 | 1,183 |
|  | 28S | 3 | 185.33 | 556 |
|  | 5.8S | 0 | 0 | 0 |
|  | 5S | 0 | 0 | 0 |
| snRNA | snRNA | 80 | 167.01 | 13,361 |
|  | CD-box | 17 | 152.35 | 2,590 |
|  | HACA-box | 16 | 204 | 3,264 |
|  | splicing | 24 | 156.21 | 3,749 |
|  | scaRNA | 21 | 165.05 | 3,466 |
|  | Unknown | 2 | 146 | 292 |

**Table S9 Statistics of closely related species gene structure of *S. mosellana***

| Species | Number | Average transcript length(bp) | Average CDS length(bp) | Average exons per gene | Average exon length(bp) | Average intron length(bp) |
| --- | --- | --- | --- | --- | --- | --- |
|  |  |  |  |  |  |  |
| Smo | 12,269 | 5,599.50 | 1,520.74 | 5.18 | 293.84 | 976.86 |
| Aae | 15,698 | 16,052.06 | 1,439.73 | 3.89 | 369.97 | 5,053.52 |
| Ban | 13,101 | 2,528.54 | 1,372.18 | 4.29 | 320.02 | 351.71 |
| Cqu | 18,968 | 5,613.00 | 1,313.53 | 3.74 | 351.22 | 1,569.22 |
| Aga | 12,959 | 5,559.81 | 1,619.37 | 4.05 | 400.02 | 1,292.70 |
| Bdo | 11,676 | 9,089.97 | 1,625.90 | 4.51 | 360.65 | 2,127.59 |
| Mde | 22,635 | 2,590.92 | 1,237.78 | 4.85 | 255.17 | 351.38 |
| Dme | 13,462 | 4,334.54 | 1,597.59 | 3.94 | 405.99 | 932.52 |

Notes: *Sitodiplosis mosellana* (Smo), *Aedes aegypti* (Aae), *Culex quinquefasciatus* (Cqu), *Anopheles gambiae* (Aga), *Belgica Antarctica* (Ban), *Mayetiola destructor* (Mde), *Drosophila melanogaster* (Dme), *Bactrocera dorsalis* (Bdo)

|  | Gene set | Number | Average transcript length(bp) | Average CDS length(bp) | Average exons per gene | Average exon length(bp) | Average intron length(bp) |
| --- | --- | --- | --- | --- | --- | --- | --- |
| novo | Augustus | 12,164 | 4,412.26 | 1,592.77 | 5.11 | 311.83 | 686.38 |
|  | GlimmerHMM | 22,410 | 6,794.18 | 939.55 | 3.69 | 254.84 | 2,179.00 |
|  | SNAP | 16,059 | 4,297.84 | 960.34 | 4.10 | 233.96 | 1,074.99 |
|  | Geneid | 19,407 | 5,223.57 | 1,147.82 | 3.58 | 320.93 | 1,581.87 |
|  | Genscan | 17,337 | 6,125.02 | 1,310.21 | 4.48 | 292.54 | 1,384.09 |
| Homolog | Dme | 7,138 | 3,764.34 | 1,351.15 | 4.42 | 305.86 | 706.13 |
|  | Mde | 10,042 | 3,145.06 | 1,400.64 | 4.45 | 314.68 | 505.48 |
|  | Bdo | 7,144 | 3,190.49 | 1,286.65 | 4.14 | 310.70 | 606.10 |
|  | Cqu | 8,496 | 2,586.33 | 1,196.17 | 3.88 | 308.23 | 482.57 |
|  | Aga | 7,578 | 3,725.76 | 1,344.15 | 4.48 | 299.97 | 684.17 |
|  | Ban | 7,232 | 3,271.03 | 1,300.91 | 4.22 | 308.44 | 612.29 |
|  | Aae | 8,042 | 3,199.87 | 1,312.54 | 4.30 | 305.21 | 571.84 |
| RNAseq | PASA | 105,860 | 6,062.84 | 1,500.70 | 5.31 | 282.73 | 1,059.01 |
|  | Transcripts | 37,115 | 14,431.06 | 4,022.84 | 7.52 | 534.67 | 1,595.40 |
| EVM | | 14,281 | 5,143.80 | 1,416.81 | 4.82 | 293.92 | 975.55 |
| Pasa-update* | | 14,129 | 5,216.90 | 1,449.78 | 4.89 | 296.68 | 969.22 |
| Final set* | | 12,269 | 5,599.50 | 1,520.74 | 5.18 | 293.84 | 976.86 |

**Table S10 Statistics of gene structure prediction of *S. mosellana* genome**

**Table S11 Gene number used for gene family clustering in each species**

| \| Symbol \| \| --- \| | \| Scientific Name \| \| --- \| | \| Gene number \| \| --- \| | |
| --- | --- | --- | --- | --- | --- | --- |
| [Smo](file:///D:\Experiment%20data\Genome%20sequencing\Report-%E9%BA%A6%E7%BA%A2%E5%90%B8%E6%B5%86%E8%99%AB%E6%AF%94%E8%BE%83%E5%9F%BA%E5%9B%A0%E7%BB%84-\Report-X101SC19090409-Z01\src\html\-) | *Sitodiplosis mosellana* | 12268 |  |
| [Cna](https://ftp.ncbi.nlm.nih.gov/genomes/all/annotation_releases/265458/100/GCF_009176525.2_AAFC_CNas_1.1/) | *Contarinia nasturtii* | 14853 |  |
| [Aae](https://www.ncbi.nlm.nih.gov/nuccore/AAGE00000000) | *Aedes aegypti* | 15712 |  |
| [Cqu](https://www.ncbi.nlm.nih.gov/nuccore/AAWU00000000) | *Culex quinquefasciatus* | 18849 |  |
| [Aga](https://www.ncbi.nlm.nih.gov/genome/?term=Anopheles+gambiae) | *Anopheles gambiae* | 12961 |  |
| [Ban](https://www.ncbi.nlm.nih.gov/genome/14659?genome_assembly_id=212588) | *Belgica antarctica* | 13105 |  |
| [Pst](http://gigadb.org/dataset/100256) | *Parochlus steinenii* | 13451 |  |
| [Mde](https://www.ncbi.nlm.nih.gov/genome/2619?genome_assembly_id=34006) | *Mayetiola destructor* | 17451 |  |
| [Dmo](https://ftp.ncbi.nlm.nih.gov/genomes/all/annotation_releases/7230/101/) | *Drosophila mojavensis* | 13168 |  |
| [Dme](https://www.ncbi.nlm.nih.gov/genome/47?genome_assembly_id=204923) | *Drosophila melanogaster* | 13472 |  |
| [Bdo](https://www.ncbi.nlm.nih.gov/genome/?term=bactrocera+dorsalis) | *Bactrocera dorsalis* | 12103 |  |
| [Bmo](http://metazoa.ensembl.org/Bombyx_mori/Info/Index) | *Bombyx mori* | 13259 |  |
| [Tca](https://pubmed.ncbi.nlm.nih.gov/18362917/) | *Tribolium castaneum* | 12799 |  |
| [Nvi](http://metazoa.ensembl.org/Nasonia_vitripennis/Info/Index) | *Nasonia vitripennis* | 13573 |  |
| [Dpu](http://metazoa.ensembl.org/Daphnia_pulex/Info/Annotation/#assembly) | *Daphnia pulex* | 30587 |  |

Notes: *Sitodiplosis mosellana* (Smo), *Contarinia nasturtii* (Cna), *Aedes aegypti* (Aae), *Culex quinquefasciatus* (Cqu), *Anopheles gambiae* (Aga), *Belgica Antarctica* (Ban), *Parochlus steinenii* (Pst), *Mayetiola destructor* (Mde), *Drosophila mojavensis* (Dmo), *Drosophila melanogaster* (Dme), *Bactrocera dorsalis* (Bdo), *Bombyx mori* (Bmo), *Tribolium castaneum* (Tca), *Nasonia vitripennis* (Nvi), *Daphnia pulex* (Dpu)

**Table S12 The distribution of genes in different species**

| Species | Single-copy genes | Multiple-copy genes | Unique | Other | Uncluster |
| --- | --- | --- | --- | --- | --- |
| [Smo](file:///D:\Experiment%20data\Genome%20sequencing\Report-%E9%BA%A6%E7%BA%A2%E5%90%B8%E6%B5%86%E8%99%AB%E6%AF%94%E8%BE%83%E5%9F%BA%E5%9B%A0%E7%BB%84-\Report-X101SC19090409-Z01\src\html\-) | 2097 | 926 | 375 | 7101 | 1769 |
| [Cna](https://ftp.ncbi.nlm.nih.gov/genomes/all/annotation_releases/265458/100/GCF_009176525.2_AAFC_CNas_1.1/) | 2103 | 1032 | 1387 | 8939 | 1392 |
| [Aae](https://www.ncbi.nlm.nih.gov/nuccore/AAGE00000000) | 1925 | 1446 | 672 | 10040 | 1629 |
| [Cqu](https://www.ncbi.nlm.nih.gov/nuccore/AAWU00000000) | 1909 | 1472 | 1875 | 10832 | 2761 |
| [Aga](https://www.ncbi.nlm.nih.gov/genome/?term=Anopheles+gambiae) | 2144 | 878 | 653 | 8141 | 1145 |
| [Ban](https://www.ncbi.nlm.nih.gov/genome/14659?genome_assembly_id=212588) | 2126 | 876 | 579 | 7095 | 2429 |
| [Pst](http://gigadb.org/dataset/100256) | 1996 | 1180 | 1080 | 6773 | 2422 |
| [Mde](https://www.ncbi.nlm.nih.gov/genome/2619?genome_assembly_id=34006) | 2097 | 902 | 2174 | 8081 | 4197 |
| [Dmo](https://ftp.ncbi.nlm.nih.gov/genomes/all/annotation_releases/7230/101/) | 2168 | 808 | 261 | 9028 | 903 |
| [Dme](https://www.ncbi.nlm.nih.gov/genome/47?genome_assembly_id=204923) | 2183 | 762 | 397 | 8887 | 1243 |
| [Bdo](https://www.ncbi.nlm.nih.gov/genome/?term=bactrocera+dorsalis) | 2215 | 661 | 471 | 7470 | 1286 |
| [Bmo](http://metazoa.ensembl.org/Bombyx_mori/Info/Index) | 2212 | 688 | 1406 | 6572 | 2381 |
| [Tca](https://pubmed.ncbi.nlm.nih.gov/18362917/) | 2186 | 823 | 1105 | 7275 | 1410 |
| [Nvi](http://metazoa.ensembl.org/Nasonia_vitripennis/Info/Index) | 2188 | 752 | 1950 | 7008 | 1675 |
| [Dpu](http://metazoa.ensembl.org/Daphnia_pulex/Info/Annotation/#assembly) | 2036 | 1632 | 11871 | 8017 | 7031 |

**Table S13 GO enrichment analysis of contracted genes in *S. mosellana* (p-adjusted<0.05)**

| **GO ID** | **Description** | **out** | **All** | **pvalue** | **p-adjust** |
| --- | --- | --- | --- | --- | --- |
| **# Biological Process** | |  |  |  |  |
| GO:0005975 | carbohydrate metabolic process | 4 | 164 | 0.002629 | 0.016119295 |
| GO:0055114 | oxidation-reduction process | 5 | 332 | 0.005992 | 0.033565327 |
| GO:0007608 | sensory perception of smell | 2 | 33 | 0.006189 | 0.033565327 |
| **# Cellular Component** | |  |  |  |  |
| Not significant |  |  |  |  |  |
| **# Molecular Function** | |  |  |  |  |
| GO:0042626 | ATPase activity, coupled to transmembrane movement of substances | 5 | 43 | 3.60E-07 | 1.27E-05 |
| GO:0016614 | oxidoreductase activity, acting on CH-OH group of donors | 4 | 37 | 8.16E-06 | 0.000163416 |
| GO:0017111 | nucleoside-triphosphatase activity | 8 | 318 | 1.04E-05 | 0.000163416 |
| GO:0050660 | flavin adenine dinucleotide binding | 4 | 40 | 1.12E-05 | 0.000163416 |
| GO:0003824 | catalytic activity | 19 | 2438 | 5.76E-05 | 0.000580538 |
| GO:0000166 | nucleotide binding | 10 | 799 | 0.000271 | 0.002121932 |
| GO:0043168 | anion binding | 10 | 870 | 0.000545 | 0.003838984 |
| GO:0004386 | helicase activity | 3 | 49 | 0.000691 | 0.004430732 |


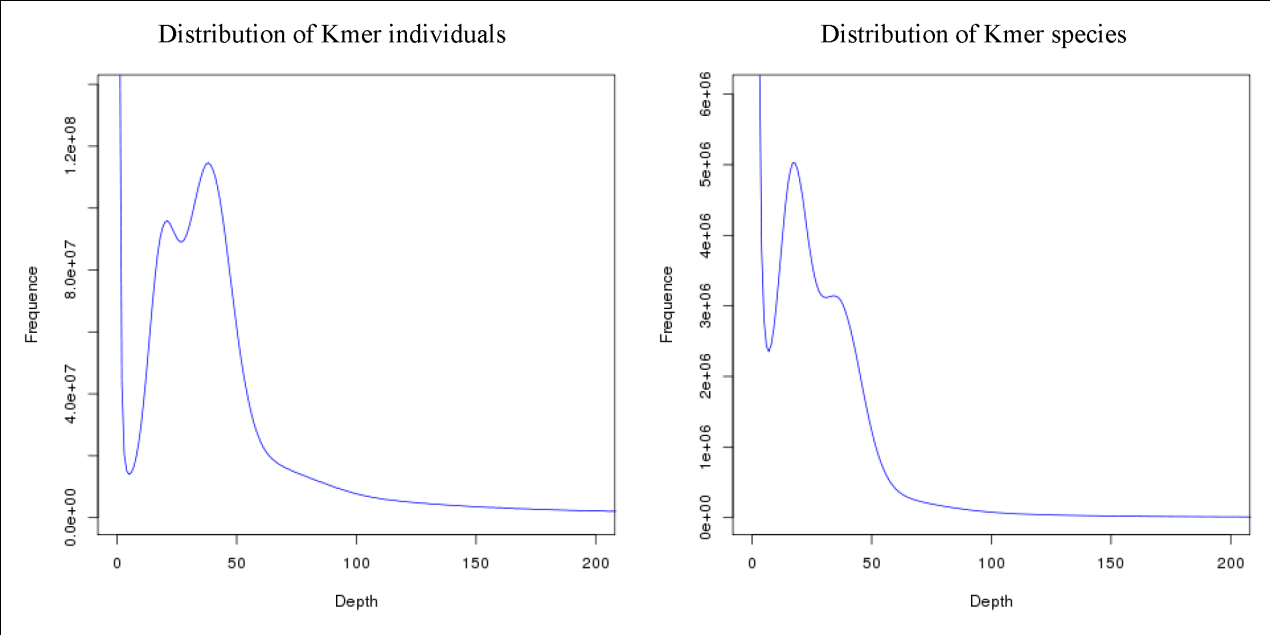


Figure S1 The K-mer analysis of genome survey of *S.mosellana*. The genome survey sequencing data were used to count of k-mers in DNA with K=17


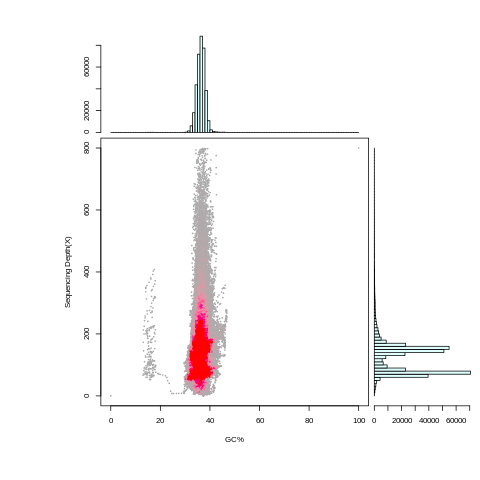


Figure S2 The distribution map of GC content and sequencing depth.

Master digram: Horizontal coordinate and vertical coordinate stand for GC content and sequencing depth respectively. The upper bar chart showed the distribution of GC content and the right bar chart showed the sequencing depth. The map showed that there is no other exogenous contamination in the genome for GC content mainly distributed in the vicinity 36.4% and scattered plot with no obvious separation.


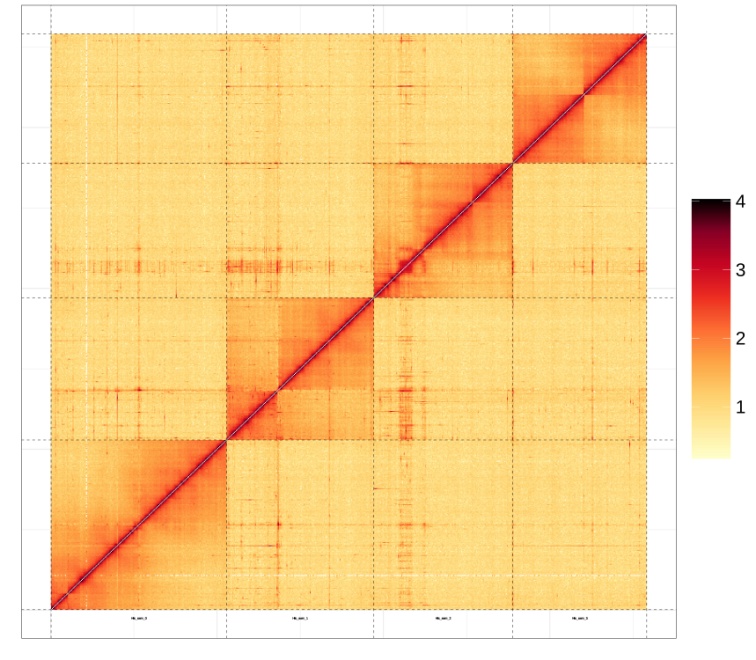


Figure S3 Heat map of Hi-C contact information of the 4 chromoseomes of *S. mosellana*. Interaction frequency distribution of Hi-C links among chromosomes showed in color key of heatmap ranging from light yellow to dark red indicating higher contact intensity.


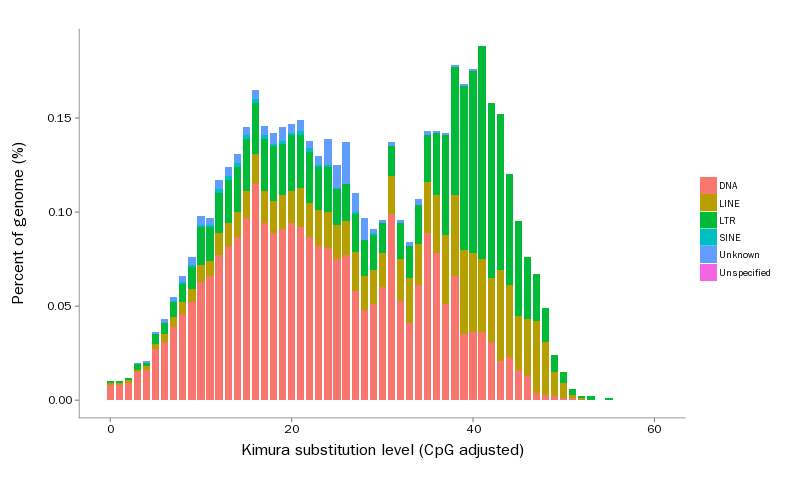


Figure S4 The distribution map of TE sequence bifurcation according to RepeatMasker.

The horizontal coordinate means the sequence bifurcation between the annotated TE sequence in *S. mosellana* genome and the corresponding sequence in the Repbase. The vertical coordinate means the percentage of TE sequence in the genome under this bifurcation. Different TE are marked with different colors. The denovo + repbase length is 37,966,242 bp accounting for 21.01 % in genome. The TE proteins length is 1,963,894 bp accounting for 1.09 % in genome. The combined TEs length is 38,105,034 bp accounting for 21.09 % in genome.


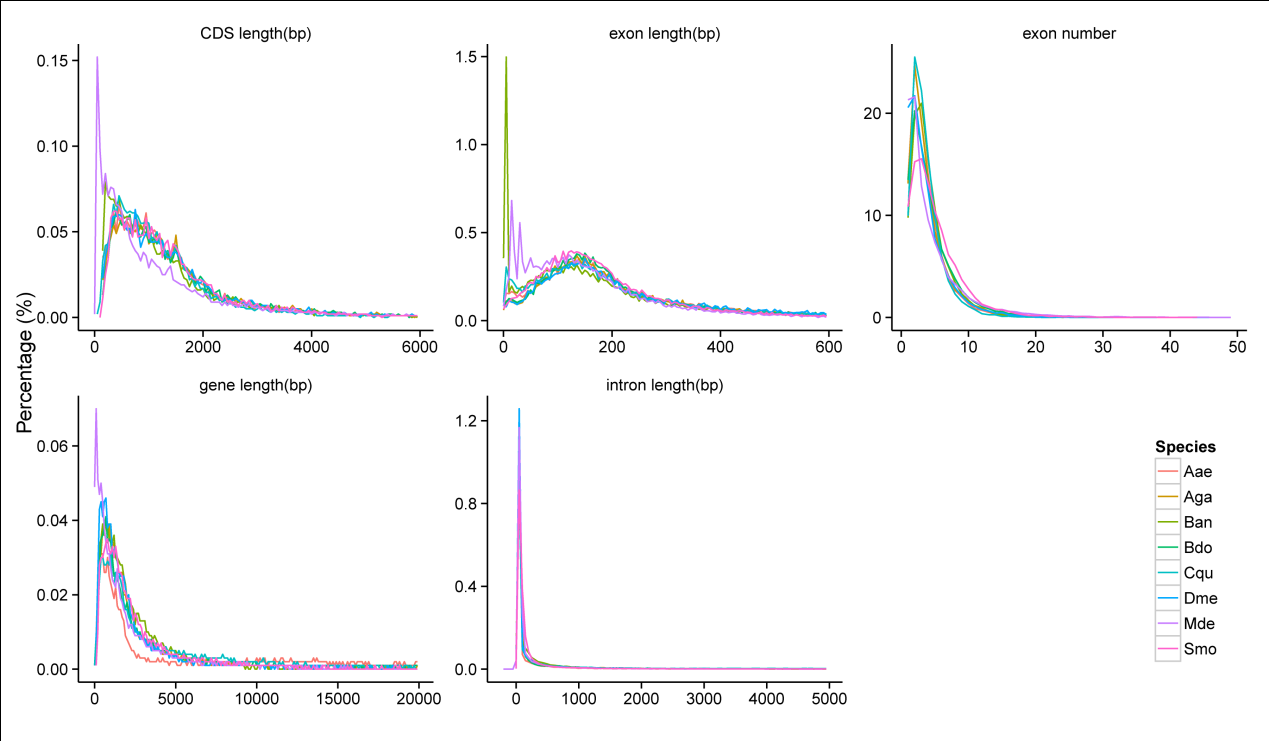


Figure S5 Comparisons of CDS length, exon length, gene length, intron length and exon number of *S. mosellana* and other closely related species genome. Species designations are: *Aedes aegypti* (Aae), *Anopheles gambiae* (Aga), *Belgica antarctica* (Ban), *Bactrocera dorsalis* (Bdo), *Culex quinquefasciatus* (Cqu), *Drosophila melanogaster* (Dme) and *Sitodiplosis mosellana* (Smo).


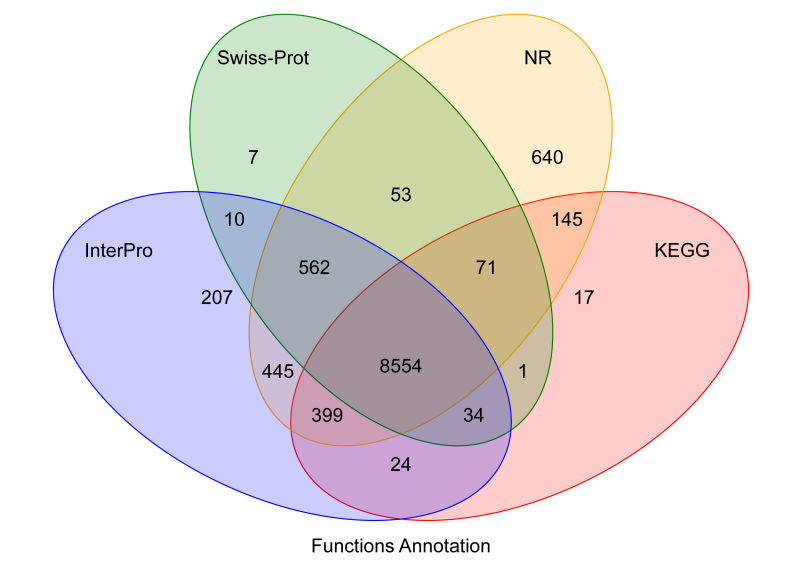


Figure S6 Venn diagram of the functional annotation. Venn diagram showing the number of genes blasted to the four databases: NR, Swiss-prot, KEEG and InterPro. Overlapped unigenes are indicated in the intersections.

**
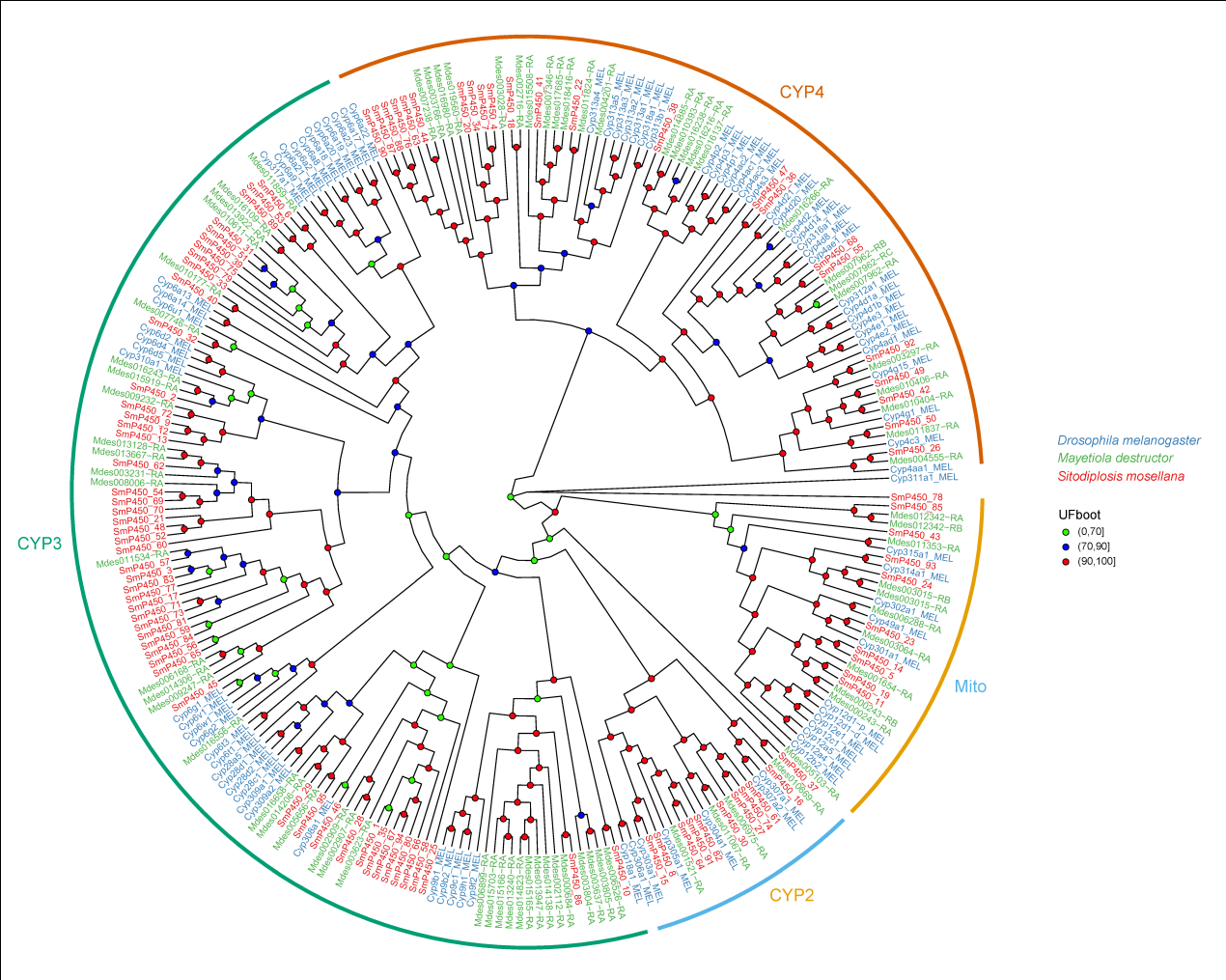
**

**FIGURE S7** Phylogenetic relationships of the Cytochrome P450 monooxygenase (P450) in *S. mosellana*. *S. mosellana* genes are labelled in red, *M. destructor* genes are labeled in green and *D. melanogaster* genes are labeled in blue. The tree was constructed using IQ-TREE and visualized by the ggtree R package.

**
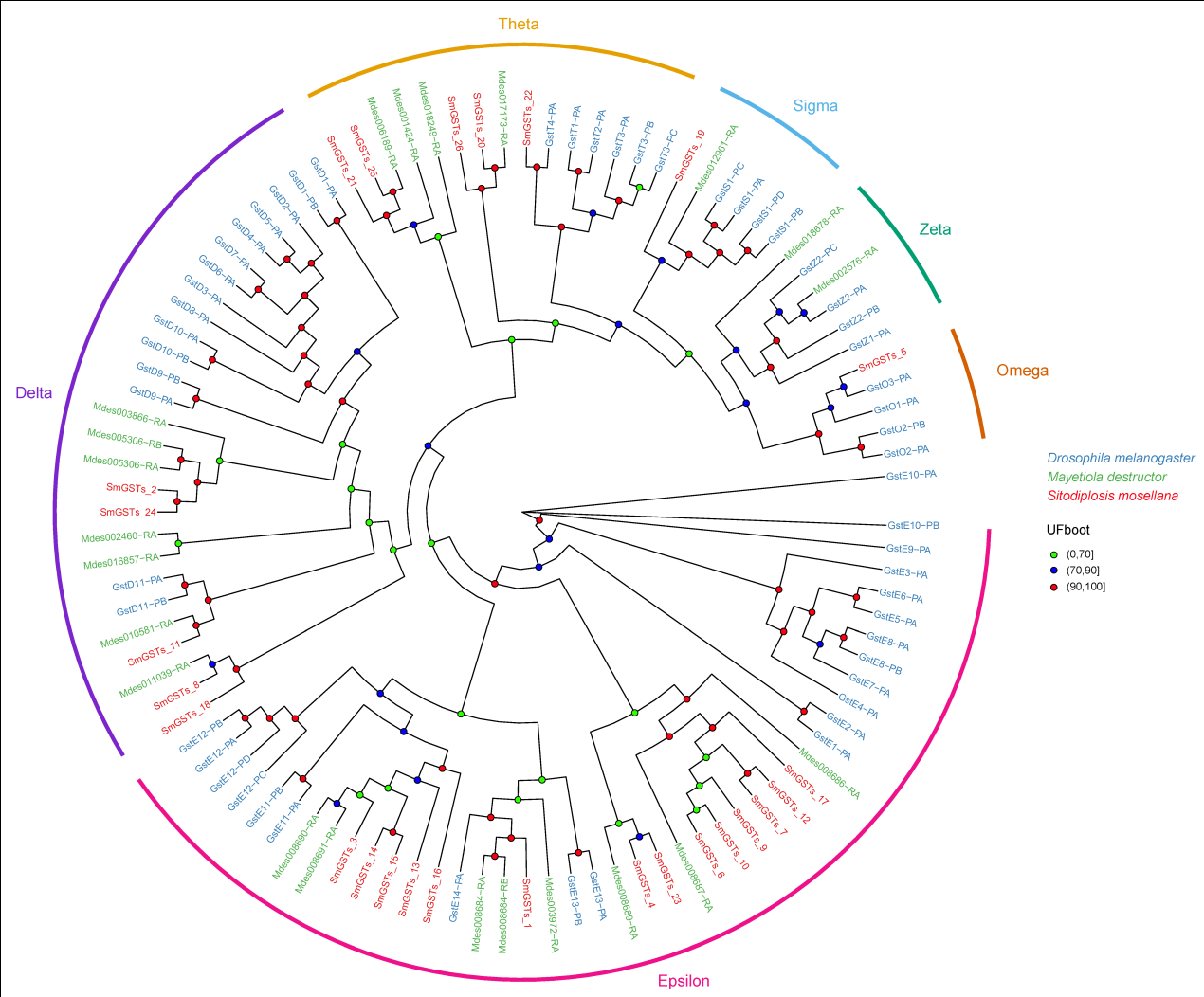
**

**FIGURE S8** Phylogenetic relationships of the glutathione S-transferase (GST) in *S. mosellana*. *S. mosellana* genes are labelled in red, *M. destructor* genes are labeled in green and *D. melanogaster* genes are labeled in blue. The tree was constructed using IQ-TREE and visualized by the ggtree R package.

**Code avilabilty**

The versions, settings and parameters of the software used in this work are as follows:

**Genome assembly:**

1. Falcon: version 1.8.2; parameters: length_cutoff_pr = 8000

job_queue= all.q

pa_concurrent_jobs = 400
cns_concurrent_jobs = 400
ovlp_concurrent_jobs = 400
pa_HPCdaligner_option =  -v -B128 -t32 -e.70  -k16 -h500 -l2000 -w8 -T8 -s1000
ovlp_HPCdaligner_option = -v -B128  -t16 -e.96 -k18 -h1080  -l1000 -w8 -T8 -s1000
pa_DBsplit_option = -x500 -s100
ovlp_DBsplit_option = -x500 -s100
falcon_sense_option = --output_multi --min_idt 0.70 --min_cov 2 --max_n_read 300 --n_core 12
falcon_sense_skip_contained = True
overlap_filtering_setting = --max_diff 100 --max_cov 150 --min_cov 2 --n_core 20

1. Quiver: version: 2.1.0; parameters: all parameters were set as default;
2. pilon: version:1.22; all parameters were set as default
3. Purge Haplotigs: all parameters were set as default;
4. ALLHiC: version: 0.9.8; parameters: --minREs 50 --maxlinkdensity 3 --NonInformativeRabio 0

**Genome annotation:**

(1) RepeatProteinMask: parameters: --noLowSimple -pvalue 0.0001 -engine ncbi.

(2) RepeatMasker: version: 4.1.0; parameters: -a -nolow -no_is -norna -parallel 4.

(3) LTR_FINDER: version:1.06; parameters: -C -w 2.

(4) RepeatModeler: version: 2.0.1; parameters-engine ncbi -pa 15.

(5) RepeatScout: version: 1.0.5; parameters: all parameters were set as default.

(6) TRF: version: 4.09; parameters: 2 7 7 80 10 50 2000 -d -h -ngs

(7) Augustus: version: 3.2.3; parameters: --species=pasa1 --uniqueGeneId=TRUE --noInFrameStop=TRUE --GFF3=on --genemodel=complete --strand=both

(8) GlimmerHMM: version: 3.0.4; parameters: -d pasa1 -f -g

(9) Genscan: version: 1.0; parameter: HumanIso.smat

(10) Geneid: version: 1.4; parameters: -P homo_sapiens.param -v -G -p geneid

(11) Genewise: version: 2.4.1; parameters: -tfor -genesf -gff -sum

(12) Interproscan: version v5.35-74.0; parameters: -cpu 20 -format tsv -appl ProDom,SMART,ProSiteProfiles,PRINTS,Pfam,Panther -iprlookup -dp -goterms

(13) EVM (EVidenceModeler): version: 1.1.1; parameters: --segmentSize 200000 --overlapSize 20000 --min_intron_length 20.

(14) PASA: version: 2.3.3; parameters: all parameters were set as default.

(15) Trinity: version 2.1.1; parameters: --normalize_reads --full_cleanup --min_glue 2 --min_kmer_cov 2 --KMER_SIZE 25.

(16) Hisat2: version 2.0.4; parameters: all parameters were set as default.

(17) Stringtie: version 1.3.3; parameters: all parameters were set as default.

**Gene family identification and phylogenetic analysis:**

1. Orthomcl: version 1.4; parameters: -mode 3 -inflation 1.5.
2. MUSCLE: version 3.8.31; parameters: all parameters were set as default.
3. RAxML: version: 8.2.12; parameterspep: -m PROTGAMMAAUTO -p 12345 -x 12345 -# 100 -f ad.
4. MCMCTREE: version 4.9; parameters: all parameters were set as default.
5. Café: version 4.2; parameters: -p 0.05 -t 4 -r 10000.
